# The emergence and current performance of a health research system: lessons from Guinea Bissau

**DOI:** 10.1186/1478-4505-10-5

**Published:** 2012-02-09

**Authors:** Maarten O Kok, Amabelia Rodrigues, Augusto Paulo Silva, Sylvia de Haan

**Affiliations:** 1 Department of Health Sciences, VU University, De Boelelaan 1085, 1081 HV Amsterdam, The Netherlands; 2 Instituto Nacional da Saúde Pública, CP 861, 1004 Bissau Cedex, Bissau. República da Guiné-Bissau; 3 Minsterio da Saúde Pública, Avenida Unidade Africana, Caixa Postal 50, 1013, Bissau Cedex, República da Guiné-Bissau; 4 Council on Health Research for Development (COHRED), 1-5 Route des Morillons, 1211 Genève, Switzerland

## Abstract

**Background:**

Little is known about how health research systems (HRS) in low-income countries emerge and evolve over time, and how this process relates to their performance. Understanding how HRSs emerge is important for the development of well functioning National Health Research Systems (NHRS). The aim of this study was to assess how the HRS in Guinea Bissau has emerged and evolved over time and how the present system functions.

**Methods:**

We used a qualitative case-study methodology to explore the emergence and current performance of the HRS, using the NHRS framework. We reviewed documents and carried out 39 in-depth interviews, ranging from health research to policy and practice stakeholders. Using an iterative approach, we undertook a thematic analysis of the data.

**Results:**

The research practices in Guinea Bissau led to the emergence of a HRS with both local and international links and strong dependencies on international partners and donors. The post-colonial, volatile and resource-dependent context, changes in donor policies, training of local researchers and nature of the research findings influenced how the HRS evolved. Research priorities have mostly been set by 'expatriate' researchers and focused on understanding and reducing child mortality. Research funding is almost exclusively provided by foreign donors and international agencies. The training of Guinean researchers started in the mid-nineties and has since reinforced the links with the health system, broadened the research agenda and enhanced local use of research. While some studies have made an important contribution to global health, the use of research within Guinea Bissau has been constrained by the weak and donor dependent health system, volatile government, top-down policies of international agencies, and the controversial nature of some of the research findings.

**Conclusions:**

In Guinea Bissau a de facto 'system' of research has emerged through research practices and co-evolving national and international research and development dynamics. If the aim of research is to contribute to local decision making, it is essential to modulate the emerged system by setting national research priorities, aligning funding, building national research capacity and linking research to decision making processes. Donors and international agencies can contribute to this process by coordinating their efforts and aligning to national priorities.

## Background

During the past decades it has become clear that research does not automatically contribute to better action for health. A first challenge is to attune research to the health needs of the population for which it is intended. Research tends to be oriented towards the interests of scientists, funders and powerful interest groups, instead of the health needs of populations and interests of more marginalized groups [1,2]. Once relevant knowledge is created, a further challenge is to improve its use [1,3]. Attempts to enhance the use of research findings have for many years focused on better disseminating and explaining the right packages of information and helping receivers unpack and understand findings [4,5]. More recent approaches stress that the impetus for using knowledge must come from the users themselves, from their conception of the situation and self identity, interpretation of research findings and capacity to act, which are influenced by local contexts and systems [6-9]. Prioritizing research and enhancing its use is difficult, but even more important in low-income countries (LIC) [2,10-13]. Traditionally, research in these countries depends on external donor funding, expatriate researchers play a prominent role, national priorities are seldom articulated and there is limited engagement of local governments, which tends to create a vicious cycle in which research is oriented away from national needs and contributes little to local action [1,14-16]. Developing a well functioning National Health Research System (NHRS) is seen as an important step for ensuring that health research is targeted to the specific needs of a country and contributes to local action [2,14,17]. A NHRS is described as a set of institutions that create, govern, manage, coordinate, demand, require, communicate and use knowledge resulting from research to improve the population's health and status [14]. Those who attempt to develop NHRSs mostly do so in countries where health research has been conducted in the past. During these research activities, a rudimentary 'system' of research may emerge. Such a 'system' comprises of patterns of interaction, institutional arrangements and mutual dependencies that emerge from research practices, funding relations and from actors that are performing roles [18]. Together, these patterns of interaction, institutional arrangements and mutual dependencies form a 'system'. The fact that a system has emerged in the past, has consequences for those who attempt to develop a NHRS for the future: they do not start with a blank sheet, but have to modulate a *de facto *health research system (HRS) in a desired direction. Note the distinction between a prescriptive system approach, which describes how a 'good' system should function (NHRS) and a research 'system' as a system-level phenomenon (HRS). System-level phenomena have their own dynamics and build on patterns of (mutual) dependencies that are shaped by institutions such as laws, rules, norms, organizational procedures, etc. When an actor or organization attempts to change a system, it is constrained by these patterns and so often change is minimal or absorbed by the system [9,19]. Since changing system-level phenomena is difficult, it is important to understand how HRSs emerge and evolve, and can be modulated towards well functioning NHRSs.

A country that has recently embarked on the process of better employing research for the health of its population is Guinea Bissau. This small West African country, with an estimated population of 1.5 million is one of the five poorest in the world. Since independence from Portugal in 1974, Guinea Bissau has experienced considerable political and military upheaval: a few years after the first multi party elections in 1994 the country fell into a civil war that ended by 2000 after which the country has seen a rapid succession of both military and civilian governments. During the civil war many health workers left the country and the health infrastructure rapidly deteriorated. Ongoing political instability, a lack of trained health workers and low government expenditure on health (estimated at 3 USD per capita in 2006) have hampered the reconstruction of the health system [20]. Though donor support seems to have been beneficial, it has also led to dependencies of the health sector on uncoordinated and rapidly changing policies of a multitude of donors and agencies [21]. In this volatile, resource-poor and dependent context, health research has been conducted since 1976. Most of the research has been conducted by, or in collaboration with, the Bandim Health Project (BHP), a Danish-Guinean research collaboration which has produced by far the largest part of the nearly 650 research publications that originate from Guinea Bissau (Pubmed indexed publications excluding commentaries). Meanwhile, capacity building efforts have been ongoing and led to over a dozen Guineans obtaining Master and PhD degrees in health research [22,23]. In 2005 the Ministry of Health (MOH) of Guinea Bissau started to explore ways in which the benefits of health research to its population could be enhanced. A first step was to diagnose the functioning of the existing HRS through the NHRS framework. The case of Guinea Bissau provides an interesting account of an emerging and evolving HRS that started with a Scandinavian 'reconnaissance' research mission in 1976 and continued with expatriate led and donor dependent research which resulted in hundreds of publications and laid the foundation for the current process of NHRS development. The combination of high quality research and a weak and widely challenged health system makes Guinea Bissau a unique case that provides the opportunity to learn more about the functioning of research and development dynamics in LIC. While the attention for the development and functioning of NHRSs in LIC is growing [24-26], very few accounts have been published on how HRSs in LIC emerge and function, and no attempts have been made to explore what it means to deal with a 'system'.

For the study presented in this article we aimed to analyze how the HRS in Guinea Bissau has emerged and evolved over time and how the present system functions. The importance of developing NHRSs is stressed in numerous World Health Assembly resolutions and in the 2004 and 2008 Ministerial Summits on Health Research [27]. Lessons about how HRSs emerge and evolve, and how their functioning can be improved are of relevance to all that seek to better employ research for health.

## Methods

We used a qualitative case-study methodology. We conducted document analyses and interviews to iteratively develop an account of the emergence and present functioning of the HRS. This was combined and enriched with a more in-depth exploration of ten research projects and of the utilization their results.

### Theoretical framework

The National Health Research System (NHRS) framework was used as an analytical focusing device (see Table 1). The notion of a NHRS was put on the agenda by the report of the 1990 Commission on Health Research for Development [2]. In the consultative meetings before the 2000 International Conference on Health Research for Development, Bangkok, the ideas were further refined. Based upon this earlier work, Pang et al developed the current NHRS framework which describes the functions, components and boundaries of a NHRS [17]. The main functions that are described are 1) providing stewardship, 2) financing, 3) creating and sustaining resources and 4) producing and utilizing research. For each of these functions, specific components are described (see Table 1). While we used the NHRS framework to focus and structure our analysis, we also explored international dynamics that influence health research and utilization at national level.

**Table 1:** Functions and components of a National Health Research System [17].

Stewardship	
	• Define and articulate vision and policy for a national health research system • Identify appropriate health research priorities and coordinate adherence to them • Set and monitor ethical standards for health research and research partnerships
**Financing**	
	• Secure research funds and allocate them accountably
**Creating and sustaining resources**	
	• Build, strengthen, and sustain the human and physical capacity to conduct, absorb, and utilize health research
**Producing and utilizing research for health**	
	• Produce scientifically valid research outputs • Translate and communicate research to inform health policy, practices, and public opinion • Promote the use of research to develop new tools (drugs, vaccines, devices, etc)

### Study sample

For this study we reviewed policy documents, research reports and publications and purposively selected 39 key informants based upon their current or former roles related to the prioritization, funding, conduct, distribution and use of health research from or in Guinea Bissau. Interviewees often had various professional roles and worked as health researcher, advisor, policy maker and/or practitioner at the BHP, MOH and its specialized programs or committees (EPI, HIV/AIDS, Cholera, Malaria), international and donor agencies (e.g. WHO, UNICEF, UNFPA, UNAIDS, French Cooperation, Danish Ministry of Foreign Affairs) and three regional health directorates (Bafata, Cacheu and Oio).

### Interviewing and data collection

The interviewing and data collection was done by two researchers (MK and SH) that were not previously involved in research in Guinea Bissau. Thirty-three interviews were conducted in Guinea Bissau, three in Denmark, and one each in Switzerland, United Kingdom and Portugal. Interviews were conducted in English, French and Portuguese and translated to English when necessary. The interviews were conducted for both the purpose of this study, and to inform the interviewees as preparation for the research priority setting process. A general topic list was developed, which was adapted for each interviewee, depending on his or her role, position and expected knowledge about the topic of interest. Throughout the study, new themes and topics emerged and were added to the topic list. To triangulate or further explore emerging themes, examples and key events, specific questions were also added to the interview list. Interviews were recorded and during the interviews notes were taken. Verbal consent was obtained for each interview and it was explicitly stated that the findings would be published, to which there were no objections. The first document analyses and twelve interviews focused on a general exploration of the functions and components of the NHRS. To explore the NHRS functions in depth, ten research projects (3 ongoing and 7 finalized) were chosen in consultation with the Guinean researchers as a diverse sample of the conducted research work that would be relevant for exploring the NHRS. Successive interviews focused mostly on these ten research projects, and on further exploring the emergence of the HRS and the functions of the NHRS. A timeline was drawn for each research project which included the funding, formulation and conduct of the research as well as the dissemination and utilization of the results. For each of these projects the linkages between the research project and the broader context and systems was systematically explored. The scientific publications from Guinea Bissau that seemed most influential in shaping how the research system evolved, and two publications about the history of the BHP, were used to structure the interviews about the historical emergence of the HRS.

### Data management and analysis

Directly after each interview, a detailed summary was prepared. These summaries were checked by listening to the interviews a second time. The parts of the interviews that were either complicated or contained parts that seemed important for the analysis, were selected and transcribed verbatim. The interview summaries, transcripts and documents were used to identify themes and key-events that were used to describe the emergence of the HRS. This was done similarly for the ten research projects. A manual coding system was used for the descriptions of the functions and components of the research system. Theme-specific groupings were developed and read, and the themes were modified or amplified. Illustrative quotations were identified to supplement the narrative description of the emergence of the HRS and functions of the NHRS. Preliminary findings were presented and discussed at two national workshops that were co-organized by INASA and the Council on Health Research for Development (COHRED) to inform the main stakeholders of research in Guinea Bissau about NHRS development and prepare for research priority setting. Those involved in the discussions confirmed the presented results.

## Results

In this section we first describe the emergence of the health research system in Guinea Bissau from independence in 1974 until 2010. In the second part of the result section, we describe the functioning of the HRS in 2010, using the NHRS framework.

### The emergence of health research in Guinea Bissau since independence

In 1974 Guinea Bissau became independent after a violent liberation war. The country started without any Guineans trained in health research and with a complete lack of research infrastructure. The first research activity in Guinea Bissau was a reconnaissance mission sent to the Oio region in 1976, after which the Swedish Agency for Research Cooperation (SAREC) with developing countries, decided to send an interdisciplinary team to Guinea-Bissau in 1978 for a one year project to examine the nutritional situation in the country and to suggest ways of improving nutrition and reducing child mortality. The scientists, research questions and underlying assumptions (e.g. poor nutrition is the cause of ill health), research practices and funding all came from abroad (Northern Europe). In the same year the Swedish development organization SIDA established the national laboratory for public health (Portuguese acronym: LNSP) in Bissau which performed analyses for medical care and for research. During 18 months, the project collected data in Bandim 1, an urban district in the capital of Guinea-Bissau, and in five rural areas. Research assistants were trained to collect data on births and deaths and a registration system was set up. The collected data showed that the children were not severely malnourished as was widely assumed in the North, but childhood mortality was 400-500/1000. In spite of relatively good nutritional status, measles infection had a case fatality rate of 21% in a large epidemic in 1979. After the project ended Peter Aaby, one of the original researchers, decided to continue working in the country and became the lead researcher of the Bandim Health Project (BHP). A system of continuing data collection was set up to get a better understanding of the causes of high childhood mortality. The data collected showed that the high measles mortality depended on crowding, intensive exposure, and dose of infection [22]. From 1981 measles vaccine was being introduced as a new policy and closely studied throughout the introduction. The introduction of measles vaccines led to a rapid reduction of childhood mortality from 400-500/1000 to 150-200/1000 in the study areas.

From 1983 to 1988 research remained externally funded (Danish Church Aid and DANIDA) and the focus continued to be child mortality. The basic infrastructure for data collection was maintained. A mobile team of nutritional assistants visited villages in the interior of the country for data collection, health education, as well as routine vaccinations for measles vaccine, Diphtheria, Tetanus and Pertussis (DTP) and Oral Polio Vaccine (OPV), combining both health provision and research. In addition, vaccination outreach activities in Bissau city were developed; giving health centers the responsibility for providing vaccinations for the whole of the capital. The BHP was closely linked to local health care provision activities. Vaccination coverage increased markedly in the process and after measles came under control due to the regular vaccination actions, the BHP started studies focusing on diarrhea as the next major cause of child mortality. In the same period Portuguese researchers discovered HIV 2 in samples taken from Guinea Bissau and Cape Verde [28]. Through this period collaborations were initiated with projects in The Gambia, Senegal, and Kenya to test whether the observations on measles mortality and crowding were reproducible elsewhere.

In the period from 1989 to 1993, several additional monitoring activities started. Surveillance started in the Biombo and Oio regions to monitor changes in childhood mortality. UNICEF wanted a larger survey to assess neonatal mortality in the country. The surveillance was extended to the Cacheu, Gabu and Bafata regions, covering the five largest regions, which represent 83% of the population outside the capital. An important theme in the research agenda was the growing recognition since 1989 that something was wrong with the high-titre measles vaccine (HTMV). Since BHP could follow the children who had taken part in the HTMV trials, BHP observed that girls who had received HTMV had a two-fold higher mortality than girls who had received the standard measles vaccine in both Bissau and Senegal [29,30]. These findings were brought to the attention of WHO in writing. After initially dismissing these findings WHO was persuaded through personal discussions to organize an expert meeting which decided that the findings were inconclusive and continued to recommend the use of HTMV. In 1992, the same observation was made on Haiti and after further deliberations WHO withdrew their recommendation of HTMV [31].

From the 1990s ideas about development and aid were changing and led to greater emphasis on capacity building and demand-driven approaches. In 1997 the Enhancement of Research Capacity program (ENRECA) of DANIDA funded the training of Guinean researchers (one PhD and seven MSc), which heralded a new phase in the emergence of the HRS. Most Guinean students had previously taken part in data collection or entry for the BHP and combined course training abroad with their own research projects in Guinea Bissau. This was the first graduate and postgraduate health research capacity building since independence in 1974.

The main research themes during the 1990s were inherited from previous periods including the cause and treatment of diarrhea, the role of retroviral infections, and the long-term consequences of measles infection and HTMV. Many studies attempted to pursue the implications of the previous studies on the role of the non-specific effects of vaccinations for child survival. New projects were started on, amongst others, crowding and health, cholera control, vitamin A supplementation, TB, and the impact of HIV-2. These studies came to an abrupt halt when civil war broke out in Bissau in June 1998, which lasted until 1999. During the war, BHP assumed responsibility for humanitarian aid to the numerous internally displaced persons from Bissau. Though data had been collected in connection with the humanitarian aid activities, it was only from mid-2000 that the main focus again became research and training. Through 1998 to 2002, 8 Guineans finished their masters' and doctoral studies, and became the main group of trained researchers in the health sector in Guinea-Bissau. Many of these wanted to continue research and research training. In 1998, BHP was a founding member of the INDEPTH Network, which aimed to strengthen the collaboration between longitudinal study sites across the world.

In 2000, BHP researchers published results suggesting the possibility that some of the routine vaccinations - DTP and tuberculosis vaccine BCG- might have non-specific effects on child survival [32]. The findings that vaccines had systematic non-specific and sex-differential effects were controversial since they questioned major assumptions underlying the standard intervention programs to reduce child mortality. These findings oriented the attention of the BHP researchers to organizations such as WHO that determined the health programs that were recommended to countries like Guinea Bissau. Interviewees generally felt that the international public health community has either chosen to ignore these findings or focused on refuting them, challenging the methods used and speculating about potential sources of bias [33-35]. Studies with the power to confirm these findings or explore the emerging hypotheses were not being set up [36]. The non-specific effects of vaccines and interactions of childhood interventions has remained an important theme in the research agenda of BHP since 2000.

The trends of expansion, growing Guinean involvement and diversification of the research agenda have continued since 2003. Collaboration with local institutions such as the national hospital and with the national TB and HIV programs has further reinforced links between research and the health system. The study area of the health demographic surveillance system of BHP was expanded to include a population of over 100.000 and now cover all 10 regions in the country. This expansion was partly funded by the MOH (and World Bank) with the aim to inform national policy with health indicators. An extension of the ENRECA program led to the training of an additional 4 MSc and 5 PhD students. The increasing number of Guinean researchers and growing interest in research at the MOH further strengthened links between the research system and the MOH, as illustrated by the following quote.

*"All of us (Guinean researchers), we worked before in the health system, we knew the problems that are there and we have ideas of things that are not going well. We try to think what to do. We start also creating areas of research that are closely related to the health system and that could easily be used and solve the health system problems." *(researcher/policy advisor)

In 2005 the MOH of Guinea Bissau started to explore ways in which the benefits of health research to its population could be enhanced. A senior official at the MOH approached COHRED to facilitate the process of developing the functions of a NHRS. The initial steps were to diagnose the functioning of the existing HRS through the NHRS framework, prepare a policy for health research, inform and engage stakeholders and initiate research priority setting. With support of the International Association of National Public Health Institutes (IANPHI) the Guinean Government prepared for the establishment of its own National Institute of Public Health (Portuguese acronym: INASA).

### The functioning of the health research system in 2010

In this second part of the result section, we describe the functioning of the HRS in 2010, using the NHRS framework (see table 1). First a short overview is provided, after which the separate functions and important components are described in more detail.

The functioning of the HRS in 2010 is shaped by recent capacity building efforts and institutional reorganization, combined with the practices, human resources and structures that have been developed since the mid seventies. In addition, the current HRS is influenced by the overall volatile and resource-dependent context within which it functions. With the development of INASA and the initiated priority setting process, the national government is taking a more substantial role in stewardship for health research. For financing, the research system remains almost exclusively dependent on external sources, and their willingness to align to local priorities. The training of Guinean researchers has reinforced the links with the health system, broadened the research agenda and enhanced local use of research. Despite this important progress, there are still only a few Guineans with a PhD in health research, and collaboration with external partners for training remains essential. While nearly 650 scientific publications originate from Guinea Bissau, the use of research results in the country remains limited. Interviewees described how the use of results within Guinea Bissau has been constrained by the weak and donor dependent health system, volatile government, top-down policies of international agencies, and the controversial nature of research findings. At the same time, interviewees provided some examples of the uptake of research results in health policies of the national government and locally working NGOs.

### Stewardship

The legal framework that institutionalizes health research in Guinea Bissau is provided by the Cabinet approval for INASA in 2009. INASA is designed as an independent body within the MOH and reports directly to the Minister of Health. Health research has also been integrated in the national health plan of the MOH, where research is described as essential for informing health policy, practice and innovation. Interviewees described how until recently, research topics have mostly been determined by expatriate researchers and international agencies commissioning research, and are limited by the priorities of donors and other funders.

*"For several years, research was driven by the priorities of (Danish director of BHP), what he thinks is important. We think those (priorities) are important, but we also need other research." *(researcher)

The training and increased involvement of Guineans in research since the end of the nineties has resulted in new research topics that are more closely related to national policy making (e.g. health worker salaries, quality of care).

The process of setting national research priorities is currently in progress. Representatives of all major health research, policy and practice organizations and representatives of donors and foreign agencies working in the country are engaged in this process. A first priority list is expected in 2011. The ethics committee is functioning with difficulties due to a lack of capacity and funding. At present ethical clearance for research is obtained through partner institutes and through the local ethics committee, though often with delays and additional review abroad.

### Financing

Research for health in Guinea Bissau depends on securing foreign funding sources for all costs (including salaries, equipment, supporting staff and all project costs) except for the salaries of a few Guinean INASA researchers and some supporting staff, which are funded through the MOH.

### Creating and sustaining resources

In the recently established INASA the BHP, National Public Health Laboratory, Tropical Medicine Centre (Centro de Medicina Tropical), former Department of Epidemiology (DHE), and former Information and Communication Department (DIECS) of the MOH are brought together. The BHP is a collaboration between the MOH and the Statens Serum Institute, Denmark. It currently employs 14 researchers in Guinea Bissau (7 Danish, 8 Guineans) and 8 in Denmark, over 150 research assistants and some supporting staff. The National Public Health Laboratory performs tests for various organizations in the health system and functions as the research laboratory for INASA. The country's social science institute INEP has 55 employees of whom 16 are researchers and 4 have a PhD degree. INEP has conducted various health related research projects for international agencies, such as a Knowledge, Attitude and Practice (KAP) study on water and sanitation (funded by UNICEF). In Guinea Bissau there is no university curriculum for health research and almost all Guinean health researchers have been trained through BHP and its partner institutions abroad. In total, the country now has 6 PhD-level health researchers (and 2 enrolled) and 12 master-level health researchers, which all have degrees in public health and epidemiology. No capacity has been developed in health economics, services, policy or systems research.

### Physical research infrastructure

Though a basic physical infrastructure (water pumps, generators, freezers on natural gas) for health research has been established, the poor infrastructure in the country makes this a day to day challenge. Equipment is shipped in containers from Europe to countries near Guinea Bissau, which takes a long time to arrive and is expensive. The access to scientific publications is limited to the researchers at BHP and INASA through the Portuguese version of HINARI (a programme that provides free online access to the major journals to local institutions in developing countries) and collaborating partners in Europe. Internet is available in the capital Bissau, and in 3 of the 11 regional health directorates in the countryside through unreliable phone connections.

### Producing and using health research

The number of publications indicates that by far (> 95%) the most health research in the country has been conducted through the BHP, which has focused mostly on understanding and reducing child mortality. Major research themes have been childhood infections and management, vaccines and childhood interventions. These themes have been studied through large-scale and long-term epidemiological observational studies and randomized trials, with a specific focus on non-specific and sex-differential effects of these interventions and the possible interactions between different interventions. In recent years the BHP research agenda has widened with studies on maternal health, some studies on tuberculosis and HIV prevalence and on improving hospital care in the pediatric ward. In the past, Swedish and Portuguese expatriate researchers have conducted various studies. The most significant contribution was the isolation of HIV2 by the Portuguese researchers [28]. Recently, some researchers from the UK have started research as part of a program called Effective Interventions, which focuses on lowering infant mortality rate through community based health promotion in the Tombali and Quinara District. In addition, an Italian research group from the University of Padova is conducting a collaborative study on malaria prevention together with the National Reference Hospital for Tuberculosis and Lung Disease in the capital Bissau, and a Brazilian and Portuguese collaboration is conducting a research study on traditional medicine.

### Efforts to enhance research use

Interviewees described different approaches to enhancing the use of research results in Guinea Bissau. Traditional structures for disseminating research results are very limited: there is only one scientific journal in the country which features social science. There has been one special edition on health research, which seems the only national health research publication in the country since independence [37]. Interviewed health workers at the MOH and in the regions were aware that research was conducted in the country, but were knew little about the research themes and topics of the conducted research. Until 2004 research papers were translated from English to Portuguese and disseminated locally to relevant health officials. According to interviewees, this has been halted because the translation was costly and the capacity to interpret these papers was limited to a few people in the country that were also proficient in English. Until 2008, BHP researchers have organized a number of dissemination meetings at which researchers presented their findings and discussed them with decision makers. Since then, there has not been an organized approach to disseminate research results in the country, and dissemination depends on the initiative of individual researchers.

Interviewees described that the most effective strategy for enhancing the use of research results in Guinea Bissau is personal interaction between researchers and policy makers and officials from MOH, NGOs, international agencies, and advocacy by researchers. All Guinean researchers also have tasks in the health system such as medical doctor, nurse, or advisor in boards and on *ad hoc *committees.

"*The Guineans, we are really inside (the health system). Even today if I want to discuss something with the Malaria director, I go and discuss. For vaccination (...) is in the committee. For the strategic national plan we were consulted. If we want to see the Director General or Deputy Minister, there is no problem, they are interested." *(researcher/policy advisor)

These interactions were deemed effective because the meaning of research findings can be explored through dialogue in the context of decision making. The small size of the health policy and research communities has also helped to establish trust which is important for discussing difficult findings such as the over-diagnosis of malaria or asking for illegal user fees to patients.

The use of research findings at the national level is constrained by the weak status and lack of absorptive capacity of the health system, the prominent role played by international agencies and NGOs and the nature of the research.

The country's health system functions with very few resources and a shortage of health workers within a politically instable context, which has made it difficult for the government to realize improvements based on research findings. International agencies and NGOs have stepped in to organize and support health services and provide the health system with advice, targeted funding and other support. These organizations have enhanced the use of research and innovations from other countries by stimulating the use of protocols, guidelines and new treatments. In addition, international agencies and NGOs have also commissioned some local studies (such as KAP studies on water and sanitation) that were used by themselves and the MOH, amongst others. The local opportunities of health professionals to act on research findings often depends on the perceptions, funding, advice and policies of international agencies. Though international agencies and NGOs have in some cases used local findings, the interviewees described several cases in which international agencies and NGOs failed to inform themselves of the locally conducted research or pushed their own protocols and practices despite local findings that indicated that other strategies might be more beneficial. An example is the interference of international agencies when the Guinean researchers promoted the use of their findings that showed the protective effect of limejuice in food and water against cholera [38]. Interviewees described how international agencies working in Guinea Bissau have pressed the MOH several times to not use these findings during a cholera outbreak and promoted their own guidelines instead. Requests by the local researchers for a meeting to discuss these findings and share arguments have remained unanswered.

Another factor that has made it more difficult to contribute with research to local decision making is the nature of some of the key research findings from BHP. Since the beginning, their research findings have challenged established global health assumptions and policies. Research studies showed that infectious diseases and not malnourishment were the major cause for child mortality in Guinea Bissau and that vaccination against measles was not only possible, but also protected against more than just measles [39,40]. Their studies on the effect of the WHO recommended High Titer Measles Vaccine showed increased mortality and led, together with later studies, to the international withdrawal of the vaccine [31]. Research findings from the BHP have continued to challenge established global health policies with growing evidence on the non-specific effects of routine vaccinations and vitamin A supplementation [41,42]. Though these findings have large public health implications, publishing them has not been enough to have these findings openly debated and the implications explored, let alone influence global health policies. Interviewees described that advocacy efforts such as writing letters and personally initiating meetings have been necessary to even get findings considered. The intense entanglement in the global health community of research funders, policy makers and donors is described by interviewees as a major cause for this.

"*There is only one structure. It is the same structure which is funding the policy, deciding the policy, funding the research and training people to do the same thing as funders want them to do. There is no alternative, there is no public space. A free space to learn does not exist. This is a monolithic structure and the same thing will be reinforced."*(researcher)

Despite these challenges there are many examples in which research results were used to contribute to health policy and practice in the country. Measles vaccination became national policy after it was shown to be effective, studies on HIV led to better targeting of prevention activities, the recommendation to use lime juice is currently used and recently the salaries of health workers have been increased after a study showed that this would improve performance [43].

## Discussion

The aim of this study was to analyze how the HRS in Guinea Bissau has emerged and evolved over time and how the present system functions. Our findings provide analytical insights and lessons for those who attempt to better employ research for health in LIC.

Research practices and the systems in which they take place co-evolve over time [18]. In the emergence of the HRS in Guinea Bissau, this co-evolution (or mutual influencing) can be seen in two ways. A first way is the influence of the international research and development dynamics on local research. International funding, priorities, training, knowledge transfer, trends and controversies have oriented the conducted research to those aspects of health that are of international interest. The results of some of this research have been used extensively at international level and an important contribution has been realized to international scientific progress and global health. At the same time, there has been much less attention for research questions that are specific and essential for local decision making. This includes the local research that is necessary to safely and efficiently employ internationally developed innovations (with vaccine safety research as the exception).

Countries like Guinea Bissau have little influence on international research and development dynamics, but can attempt to attune the available support to local needs and demands. The MOH has therefore started to develop a research policy and the recently established INASA is coordinating the process of setting national research priorities. Developing these and other functions of a NHRS aims to stimulate research that is attuned to local needs and contributes to local decision making. The challenge is to develop such a nationally oriented research stream in addition to the internationally oriented stream, and explore opportunities for mutual reinforcement.

To contribute with research to better action for health, alignment needs to be created between research and relevant decision making processes. Personal interaction between researchers and influential users is often an effective step towards such alignment. To develop effective personal linkages, one needs to determine at which level (local, national, international) and by whom (practitioners, MOH, international agencies, NGOs, etc) relevant decisions are actually made. Our findings indicate that some far reaching decisions are made at international level (e.g. on the vaccine schedule, cholera guidelines) and are practically imposed on the country. Our results indicate that the opportunity of Guinean health officials to use results from local research is severely constrained by the dependency on external resources, donor policies and technical advice. This worrying dynamic is likely to occur in other LIC as well and requires attention. International organizations that fund research, provide technical advice or promote health interventions in vulnerable LIC should be more aware of the local dynamics they create. Together they influence what research is being conducted and which research results are being used. An important step forward is to respect and support the strengthening of the role of the national government, as was agreed upon in the Paris declaration and Accra Agenda for Action [44].

The second way co-evolution of research practices and systems has taken place is *within *Guinea Bissau. Our findings show that during the past decades, a rudimentary 'system' of research has emerged through the research practices within the country. While this system has many international dependencies, local patterns of interactions and institutions have also emerged. Since a (rudimentary) system has emerged, subsequent efforts to develop a NHRS are best understood as attempts to modulate a *de facto *HRS in a desired direction. By taking into account system level phenomena (e.g. endogenous evolution, resiliency to change), involved actors can increase the likelihood that NHRS development succeeds [18].

Ultimately, to achieve a sustainable NHRS a continuous dynamic has to be realized *within *the country through which local priorities and funding leads to local research that contributes to local action. Only the national government can realize such a system. Our findings suggest that attempts by a government to develop a well functioning NHRS may actually be constrained by international research and development influences. It is therefore crucial that the international community enables the emergence of a well functioning NHRS by aligning funding and capacity building to national priorities, stimulating local investment in research and providing demand driven technical assistance.

Besides the importance of NHRS development, this study also points to some weaknesses in the NHRS approach. The focus on the national level is appropriate for only some of the many heterogeneous routes through which research contributes to action. More attention is needed for the linkages to research dynamics and decision making at the more local (e.g. district health directorate) and international level, and other ways of informal and organized learning such as monitoring and evaluation.

Another shortcoming of the NHRS framework is the lack of explication of *how *research is to contribute to better action for health. 'Research use' is described as one of the functions of a NHRS, but the framework does not indicate *how *research results are to contribute to better action for health, and provides little guidance on *what could be done *to strengthen the realization of such contributions. The examples of research use in Guinea Bissau indicate that these processes are more complex than often described, but also provide lessons for enhancing research use. Targeted engagement of potential end-users in the formulation, conduct and interpretation of research, sustainable interaction, trust, and active engagement of researchers in health decision making processes, all seem to have enhanced the use of results to contribute to action. Similar patterns have been found in recent case studies [45-48] that indicate that the contribution of knowledge to action ultimately depends on the end-users and the contexts in which they function. These shortcomings suggest that a more refined systems approach could be useful to better understand the link between the production of knowledge and the use of knowledge to contribute to better action for health.

In Guinea Bissau, the development of some national research capacity (through BHP and ENRECA) provided the foundation to start NHRS development. NHRS development now seems important for further attuning research capacity development to local needs. Just as in other countries in the region, capacity development will remain dependent on South-South and North-South collaboration. Further developing collaboration and networks within the region might be a step forward.

### Concerns about the functioning of the international research system

The final consideration that follows from our diagnosis is that in addition to better aligning research to national needs, a stream of research is needed that is more independent from established global health funders and policies. The research conducted at the BHP and the contributions it has already made to global health show how essential it is to have independent researchers following controversial findings and be able to challenge dominant views and policies. In multiple interviews and in various published commentaries, concern is expressed for the lack of an open scientific debate about some of the more controversial research findings, such as the positive and negative non-specific effects of vaccines [36,49-51]. Though these issues require more detailed analyses, our findings indicate that donors, policy makers and research funders in the global health community are closely entangled and have attempted to mediate the controversial research findings away, instead of welcoming them as an incentive to learn. This raises questions about the ability of the global health community to productively deal with scientific controversy. This indicates that in addition to better aligning research at the national level, arrangements for more independent critical research and debate also requires attention. If combined, these points lead us to call for a more heterogeneous architecture for health research, with a stream of research that is better aligned and embedded within NHRSs and a stream that is more independent and less entangled with international (donor) policies.

This study focused on one country and so the extent to which the findings may be reproduced in other countries is uncertain. However the study included a large sample, with a wide range of actors involved in research and policy in Guinea Bissau and at the international level. It is hoped that the detailed exploration presented here can be used to further develop our understanding and modulation of the emergence and functioning of health research systems in other contexts.

## Conclusion

In Guinea Bissau a *de facto *'system' of research has emerged through research practices and co-evolving national and international research and development dynamics. If the aim of research is to contribute to local decision making, it is essential to develop the functions of a NHRS. This means modulating the emerged system by setting national research priorities, aligning funding, building national research capacity and linking research to decision making processes. Donors and international agencies can contribute to this process by giving technical support, coordinating their efforts and aligning to national priorities.

## Competing interests

The authors declare that they have no competing interests.

## Authors' contributions

MK participated in the conception and design of the study, data collection and analysis and drafted and revised the manuscript; AR participated in the conception and design of the study, data collection, and commented on the manuscript; AS participated in the conception and design of the study and commented on the manuscript; SH participated in the conception and design of the study, data collection and analysis and the drafting and revision of the manuscript. All authors read and approved the final manuscript.
